# BarcodeBERT: transformers for biodiversity analyses

**DOI:** 10.1093/bioadv/vbag054

**Published:** 2026-02-19

**Authors:** Pablo Millan Arias, Niousha Sadjadi, Monireh Safari, ZeMing Gong, Austin T Wang, Joakim Bruslund Haurum, Iuliia Zarubiieva, Dirk Steinke, Lila Kari, Angel X Chang, Scott C Lowe, Graham W Taylor

**Affiliations:** David R. Cheriton School of Computer Science, University of Waterloo, 200 University Avenue W, Waterloo, ON N2L 3G1, Canada; David R. Cheriton School of Computer Science, University of Waterloo, 200 University Avenue W, Waterloo, ON N2L 3G1, Canada; David R. Cheriton School of Computer Science, University of Waterloo, 200 University Avenue W, Waterloo, ON N2L 3G1, Canada; School of Computing Science, Simon Fraser University, 8888 University Dr W, Burnaby BC V5A 1S6, Canada; School of Computing Science, Simon Fraser University, 8888 University Dr W, Burnaby BC V5A 1S6, Canada; Aalborg University and Pioneer Centre for AI, Øster Voldgade 3, Copenhagen 1350, Denmark; College of Engineering, University of Guelph, 50 Stone Rd E, Guelph, ON N1G 2W1, Canada; Vector Institute, 108 College St W1140, Toronto, ON M5G 0C6, Canada; College of Engineering, University of Guelph, 50 Stone Rd E, Guelph, ON N1G 2W1, Canada; David R. Cheriton School of Computer Science, University of Waterloo, 200 University Avenue W, Waterloo, ON N2L 3G1, Canada; School of Computing Science, Simon Fraser University, 8888 University Dr W, Burnaby BC V5A 1S6, Canada; Alberta Machine Intelligence Institute (Amii), 10065 Jasper Ave #1101, Edmonton, AB T5J 1S5, Canada; Vector Institute, 108 College St W1140, Toronto, ON M5G 0C6, Canada; College of Engineering, University of Guelph, 50 Stone Rd E, Guelph, ON N1G 2W1, Canada; Vector Institute, 108 College St W1140, Toronto, ON M5G 0C6, Canada

## Abstract

**Motivation:**

In the global effort to characterize biodiversity, short species-specific genomic sequences known as DNA barcodes enable fine-grained comparisons among organisms within the same kingdom of life. Although machine learning algorithms specifically designed for the analysis of DNA barcodes are becoming more popular, most existing methodologies rely on generic supervised training algorithms.

**Results:**

We introduce BarcodeBERT, a family of models tailored to biodiversity analysis and trained exclusively on data from a reference library of 1.5 M invertebrate DNA barcodes. We evaluate BarcodeBERT on taxonomic identification tasks against a spectrum of machine learning approaches, including supervised training of classical neural architectures and fine-tuning of general DNA foundation models. Our self-supervised pretraining strategies on domain-specific data outperform fine-tuned foundation models, especially in identification tasks involving lower taxa such as genera and species. Compared with BLAST, a widely used sequence-search tool, BarcodeBERT achieves comparable species-level classification accuracy while being 55× faster. Our analysis of masking and tokenization strategies also provides practical guidance for building customized DNA language models, emphasizing the importance of aligning model training strategies with dataset characteristics and domain knowledge.

**Availability and implementation:**

The code repository is available at https://github.com/bioscan-ml/BarcodeBERT.

## 1 Introduction

The task of estimating and understanding biodiversity on our planet remains a monumental challenge, as traditional methods of taxonomic analysis often struggle to keep pace with the rate of discovery and identification of new species. In this context, the search for highly expressive, short standardized genomic regions containing meaningful taxonomic information (DNA barcodes) has become prominent in biodiversity research over the past two decades (Hebert *et al.* 2003, Miller 2007, Hebert *et al.* 2016, Srivathsan *et al.* 2021). Specifically, a 658-base-pair-long fragment of the Cytochrome c Oxidase Subunit I (COI) gene (Lunt *et al.* 1996) has emerged as the de facto DNA barcode for kingdom *Animalia* (Dopheide *et al.* 2019) and has proven effective in addressing inherent taxonomic challenges. Particularly, barcodes can be used for fast and accurate queries to categorize novel specimens into existing taxa. Furthermore, in the absence of clear species boundaries, they can be used to systematically separate specimens into groups of closely related organisms. These clusters, known as operational taxonomic units (OTUs), correspond to groups of similar specimens and can be labeled using e.g. a Barcode Index Number (BIN) (Ratnasingham and Hebert 2007). As it is defined systematically, such a BIN system overcomes ambiguities in traditional species labelling and thus accelerates biodiversity research. Among the numerous taxonomic groups to which DNA barcoding is applicable, invertebrates, particularly arthropods, stand out as an incredibly diverse and taxonomically complex group (Basset *et al.* 2012), making them the focus of many methodological studies (Badirli *et al.* 2021, 2023, Gong *et al.* 2025). The diversity and taxonomic richness of this group require specialized algorithmic approaches that can capture the taxonomic structure of the data. Consequently, biodiversity researchers are increasingly turning to machine learning methods, including convolutional neural networks (CNNs) (Badirli *et al.* 2021) and transformer models (Gong *et al.* 2025), to scale taxonomic classification of arthropods and accelerate species discovery.

Transformer-based models, pretrained at scale with self-supervised learning (SSL), also referred to as “foundation models,” have found applications across diverse domains thanks to their effectiveness in learning from large unlabeled datasets (Brown *et al.* 2020, Touvron *et al.* 2021). Such models are often task-agnostic and can perform well on a variety of downstream tasks after fine-tuning. Unlike alignment-based methods, which require computationally expensive queries against a reference database for each new application Altschul *et al.* (1990), the embeddings learned by transformer-based models can be directly applied to diverse downstream tasks. Despite their success in other domains, their application for taxonomic identification using DNA barcodes has not yet been extensively explored. Moreover, most DNA-based foundation models primarily target human chromosomal DNA sequences (Ji *et al.* 2021, Zhou *et al.* 2023, Dalla-Torre *et al.* 2023), making them suboptimal for barcode data due to domain-shift between these data types. Though short, barcodes encode rich taxonomic information across over 265 000 animal species in the Barcode of Life Data System (BOLD).

Here, we aim to unlock the potential of transformer-based architectures for taxonomic identification of arthropod barcodes, providing insights that extend beyond broad, foundation-style approaches. We address the previously mentioned issues (i.e. the taxonomic complexity of arthropods, and the lack of specialized transformer models trained on DNA barcodes) by adopting a semi-supervised learning approach, followed by fine-tuning on high-quality labeled barcode data, demonstrating the value of targeted model development for specialized applications. We propose BarcodeBERT, a self-supervised method that leverages a reference library of 1.5 M invertebrate barcodes (deWaard *et al.* 2019) and a masked language model (MLM) training strategy to effectively compute meaningful embeddings of the data, facilitating successful species-level classification of insect DNA barcodes in general scenarios. In addition to the classification of known species, our pretraining strategy enables the model to generate meaningful embeddings for new sequences from previously unseen taxa, yielding substantial improvements in fully open-world settings and in non-parametric classification at higher levels of the taxonomic hierarchy.

To summarize our contributions, we first investigate the impact of pretraining using a large and diverse DNA barcode dataset (1 million sequences, from more than 17 000 species, across 6700 genera) on generalization to other downstream tasks. Second, we compare BarcodeBERT against several baselines such as pretrained DNA foundation models: DNABERT (Ji *et al.* 2021), DNABERT-2 (Zhou *et al.* 2023), DNABERT-S (Zhou *et al.* 2025), the Nucleotide Transformer NT (Dalla-Torre *et al.* 2023), and HyenaDNA (Nguyen *et al.* 2023), a CNN baseline following the architecture introduced by Badirli *et al.* (2021), and the widely used alignment-based method BLAST (Altschul *et al.* 1990). Third, our study provides actionable insights regarding tokenization strategies, optimal masking ratios, and the importance of application-specific pretraining for DNA language models. Lastly, we note that this is the first application of deep learning methods to an extensive barcode collection of Canadian invertebrates.

Overall, BarcodeBERT outperforms all other foundation models in supervised species classification, matching BLAST’s accuracy while being 55× faster and more scalable. Moreover, a linear classifier trained on BarcodeBERT embeddings has ∼6% higher species classification accuracy than the top-performing foundation model in this task. Lastly, the same embeddings can also be used for accurate genus classification using similarity searches, outperforming the top-performing foundation model by ∼30%.

## 2 Related work

The exponential growth of genomic datasets with the advent of high-throughput sequencing has both demanded and enabled a surge in classification tools for DNA sequences. Such tools are essential for large-scale biodiversity studies, where algorithmic approaches can expedite the taxonomic categorization of novel specimens. One intuitive approach is to embed sequences into a vector space where geometric distances approximate taxonomic similarities (Corso *et al.* 2021). This allows for rapid comparisons between newly sequenced and labeled DNA, enabling accurate taxonomic assignments.

Many machine learning approaches, particularly in representation learning, have demonstrated considerable potential in biodiversity analyses as they can embed raw DNA data into an expressive lower-dimensional space. Transformer-based models (Vaswani *et al.* 2017) capture complex patterns within sequential data and have shown exceptional performance in various representation learning tasks across domains, either with or without supervision (Brown *et al.* 2020, Touvron *et al.* 2021, Caron *et al.* 2021). These models are especially effective in learning from vast unlabeled datasets, making them ideal candidates for the analysis of genomic data, where obtaining high-quality annotations remains challenging.

There has been a growing number of self-supervised learning-based DNA language models proposed recently, most of which are based on the transformer architecture and trained using the masked language model (MLM) objective. The first foundational model in this space, DNABERT, utilizes a BERT-based transformer architecture along with *k*-mer tokenization for genome sequence prediction tasks. Following DNABERT, other models have emerged, including the Nucleotide Transformer (Dalla-Torre *et al.* 2023), GENA-LM (Fishman *et al.* 2025), and HyenaDNA (Nguyen *et al.* 2023). While each model varies in architectural details, tokenization methods, and training data, their reliance on SSL and the MLM objective for pretraining remains a constant. HyenaDNA is a unique entry in this space as it uses a state-space model (SSM) based on the Hyena architecture (Poli *et al.* 2023) and trains it for next-token prediction (a causal MLM).

The landscape of machine learning models specifically tailored for DNA barcodes is less developed. A recent study (Badirli *et al.* 2023) proposes a Bayesian framework based on CNNs which, when combined with visual information, achieves high accuracies in species-level identification of seen species and genus-level inference of novel species in a dataset of ∼32 000 insect DNA barcodes. This method uses supervised learning to compute meaningful embeddings that can be used as side information in a two-layer Bayesian zero-shot learning framework. Transformer methods have been introduced for the classification of fungal Internal Transcribed Spacer sequences without any self-supervision (Romeijn *et al.* 2024).

Although there has been a growing number of SSL-based DNA language models proposed in the recent literature, our findings indicate that models pretrained on a diverse set of non-barcode DNA sequences underperform on downstream barcode tasks. This suggests that general DNA foundation models may struggle with the domain-specific characteristics of barcode data. In this study, we leverage barcode-specific training to improve both species-level classification accuracy and generalization to other taxonomic ranks. By grounding our approach in targeted data and architectural choices, we seek to advance the utility of machine learning in biodiversity research, moving beyond general off-the-shelf models trained to classify specimens into known taxa. Distinctly, our specialized models are not only capable of classifying known species but also can be used for taxonomic classification for species that are not present in the training set.

## 3 Methods

In this section, we outline the key elements of our methodology. We begin with a detailed account of our datasets and data processing pipeline. We then describe the architectures and hyperparameters used in the development of BarcodeBERT.

### 3.1 Dataset

We use a reference library for Canadian invertebrates (deWaard *et al.* 2019) for model training and testing, comprised of 1.5 M DNA samples from BOLD (Ratnasingham and Hebert 2007). The dataset was further pre-processed and subdivided as described below.

#### 3.1.1 Data pre-processing

To ensure data integrity and consistency, we performed a series of pre-processing steps over this dataset. First, empty entries were removed. Then, following standard practices (Dalla-Torre *et al.* 2023), IUPAC ambiguity codes (non-ACGT symbols), including alignment gaps, were uniformly replaced with the symbol N. Duplicate sequences were removed to avoid redundancy and increase the complexity of the training and pretraining tasks. Sequences with trailing N’s were truncated. Finally, sequences falling below 200 base pairs or exhibiting over 50% N characters were excluded. Our pre-processed dataset is available to download at https://huggingface.co/datasets/bioscan-ml/CanadianInvertebrates-ML.

#### 3.1.2 Data partitioning

**Figure 1:**
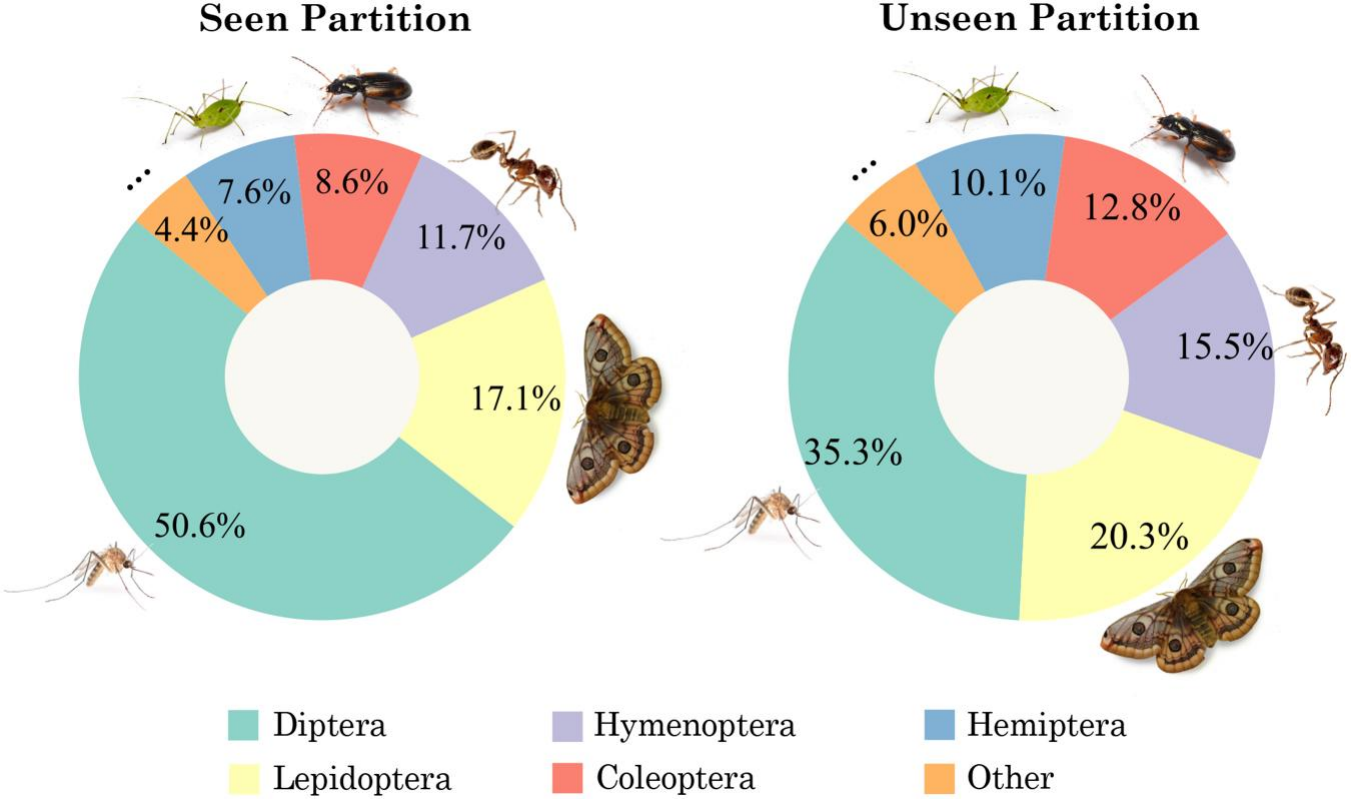
Distribution of orders in the Seen (left) and Unseen (right) partitions of the dataset. Icons: CC BY-SA, Wikimedia; Pro Content license, Canva.

After pre-processing, 965 289 barcode sequences from 17 464 invertebrate species, across 6712 genera were obtained. The dataset was divided into three distinct partitions for different training and evaluation purposes: (i) Seen: This partition is intended for supervised learning pipelines, particularly to evaluate the model’s ability to classify specimens from well-represented taxa. Comprised only of samples labeled to species-level, it includes 67 267 barcodes from 1653 arthropod species, across 500 different genera, with each species represented by at least 20 and at most 50 barcodes. The partition is further split into training (70%), testing (20%), and validation (10%) subsets. (ii) Unseen: This test partition was sampled to evaluate the models in real-world conditions where specimens from underrepresented species are frequently obtained. It only contains barcodes from “rare” species with fewer than 20 barcodes in the full reference dataset. Specifically, this partition contains 4278 barcodes from 1826 arthropod species, none of which are present in any other partition. Moreover, this partition contains all 500 genera labels present in the *Seen* partition, with up to 20 barcodes sampled per genus. The label distribution shifts are shown in Fig. 1, with the *Seen* partition reflecting the overall dataset’s distribution and the *Unseen* partition exhibiting a greater diversity of rare genera. (iii) Pretrain: This partition contains the remaining 893 744 barcode sequences from 14 794 invertebrate species across 6679 genera. Note that only 35% of the sequences in this partition contain full taxonomic annotations up to the species level. See Supplement A, available as supplementary data at *Bioinformatics Advances* online, for more details on dataset composition.

### 3.2 Proposed method: BarcodeBERT

**Figure 2:**
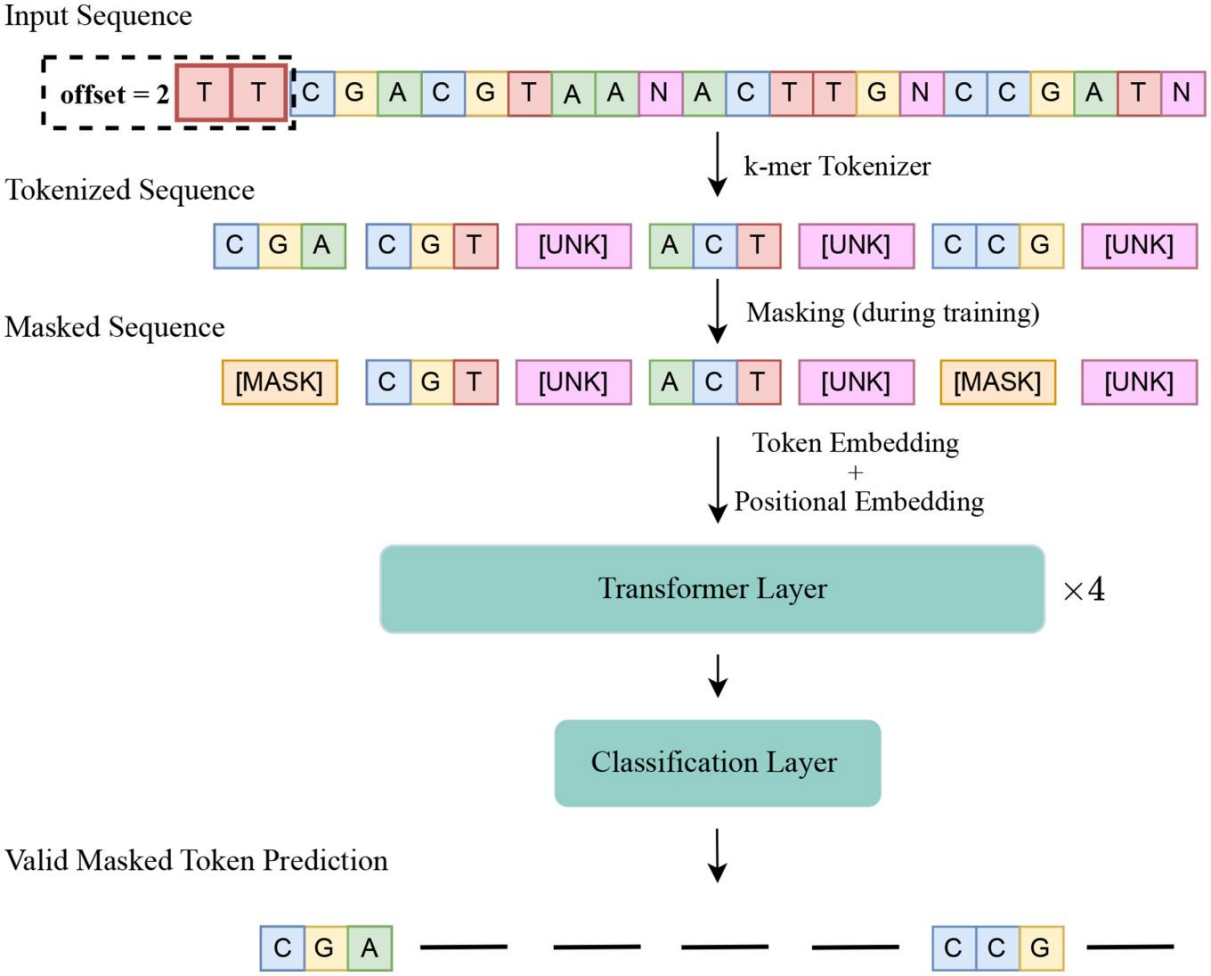
Architecture of BarcodeBERT, a transformer-based model employing a self-supervised learning strategy. The model is trained on non-overlapping *k*-mers from DNA barcode sequences. Any *k*-mer containing a character that is not in the nucleotide vocabulary is replaced by the [UNK] token. Pretraining involves masking out certain input parts using the [MASK] token and predicting these masked elements using a linear classification head. During training, the model selects a random offset (0≤ offset <k) for each sequence and begins tokenization from that position. This helps create more robust embeddings and increases resilience to potential mutations.

Inspired by Bidirectional Encoder Representations from Transformers (BERT)-like models, which convert sequence inputs into meaningful embedding vectors, BarcodeBERT is designed to encode DNA barcodes into informative embedding vectors for fast and effective comparisons. This architecture’s main building block is the transformer layer, with multi-head attention units playing a crucial role in capturing positional dependencies within each input sequence. Our model features four transformer layers, each with four attention heads, enabling a robust representation of the DNA barcode data while maintaining a manageable number of hyperparameters. Figure 2 shows the details of BarcodeBERT architecture.

Before being fed as input to the model, each barcode is split into a sequence of tokens. After evaluating two of the most common tokenization strategies for DNA sequences, Byte Pair Encoding (BPE) (Zhou *et al.* 2023, Romeijn *et al.* 2024) and *k*-mer tokenization (Ji *et al.* 2021, Dalla-Torre *et al.* 2023), we selected non-overlapping *k*-mer tokenization for BarcodeBERT (see Section 5.3 for more details). The token vocabulary includes all possible *k*-mer combinations derived from the nucleotide alphabet {A, C, G, T}, supplemented by two special tokens: [MASK] and [UNK]. The [MASK] token is utilized for masking *k*-mers during the pretraining phase, and *k*-mers containing any symbol that is not present in the nucleotide alphabet are assigned the [UNK] token. This results in a vocabulary size of $4^{k}+2$.

A limitation of this tokenization strategy is its sensitivity to frame shifts. For example, the *k*-mer representation of the sequence GATCGA differs entirely from that of CGATCGA, even though the sequences differ by only a one-nucleotide shift. To address this issue and make our model robust to frame shifts that may occur in practice, we introduce a data augmentation step by randomly offsetting the sequence by a value (0≤ offset <k) during pretraining to improve generalization. Before tokenization, DNA barcodes are either padded or truncated at 660 nucleotides to ensure coverage of the barcode region in the COI gene (Elbrecht *et al.* 2019). Finally, the tokenized sequences are fed to the model and encoded into a sequence of 768-dimensional vectors.

Following self-supervised training, our model produces a whole barcode-level embedding vector by applying global average pooling over the sequence of *d*-dimensional output vectors, ignoring padding and any special tokens. During inference, the pipeline mirrors the training setup without the random offset: DNA barcodes are tokenized into non-overlapping *k*-mers and passed through the model, generating embeddings that capture meaningful taxonomic information across the entire sequence. BarcodeBERT is implemented using PyTorch and the Hugging Face Transformers library. During training, we focused exclusively on masked token prediction, masking 50% of the input tokens and optimizing the network with a cross-entropy loss. We optimize the model parameters by gradient descent using the AdamW (Loshchilov and Hutter 2017) optimizer with weight decay set to $1\times 10^{-5}$ and a OneCycle schedule with maximum learning rate of $1\times 10^{-4}$. Additionally, we performed experiments across different *k*-mer lengths (2≤k≤8) to observe the impact of *k*-mer length on embedding quality.

## 4 Experiments

In this section, we present our evaluation framework and evaluate the performance of BarcodeBERT against the baseline models across several tasks. Additionally, we present a series of ablation studies to justify our design choices and analyze the impact of key hyperparameters on the model’s performance.

### 4.1 Experimental setup

To explore the applicability of our model for DNA barcode-based biodiversity analyses, we employ different SSL evaluation strategies (Balestriero *et al.* 2023) and contrast its performance against the baselines. First, we evaluate our models in a “closed-world” setting where the goal is to classify DNA sequences into known taxa.

#### 4.1.1 Fine-tuning

Pretrained models are fine-tuned on the training subset of the *Seen* partition and evaluated on the test subset. This task assesses the ability of models to perform species-level classification with full access to labeled training data.

#### 4.1.2 Linear probing

To evaluate the quality of pretrained embeddings, the backbone of the models is frozen, and a linear classifier is trained on the training subset of the *Seen* partition. The final classifier is evaluated on the test subset, providing insights into the effectiveness of the embeddings without extensive task-specific training.

#### 4.1.3 1-NN probing

This task evaluates model generalization to new species within known genera. Using cosine similarity, we perform 1-NN probing at the genus level with the training subset of the *Seen* partition as the reference set and the *Unseen* partition as the query set.

Second, our models are evaluated in an “open-world” setting where the goal is to group sequences, including those from unknown species, based on shared features.

#### 4.1.4 BIN reconstruction

We merge the test subset of the *Seen* partition with the *Unseen* partition and evaluate the model’s ability to reconstruct Barcode Index Numbers (BINs) using embeddings generated without fine-tuning in a zero-shot clustering (ZSC) task (Lowe *et al.* 2024). This task assesses how well the embeddings capture the hierarchical structure of taxonomic relationships, including rare or unclassified species.

Finally, we evaluate the utility of learned DNA embeddings as auxiliary information in multi-modal learning.

#### 4.1.5 Bayesian zero-shot learning

We selected a species-level image classification task using the INSECT dataset (Badirli *et al.* 2021). This is a small multimodal dataset designed for zero-shot classification of images from unseen species using DNA as auxiliary information. DNA embeddings generated by the models are paired with pre-extracted image features to classify species in a zero-shot setup. We evaluate both embeddings from pretrained and fine-tuned models on the species classification task from the INSECT dataset. Following (Badirli *et al.* 2021), the Bayesian zero-shot learning (BSZL) framework uses image features as priors and DNA embeddings as side information. For unseen species, the *K*-nearest seen species in the DNA embedding space are used to define local priors, allowing the Bayesian model to generate posterior predictive distributions for unseen categories. To ensure a fair comparison with prior work, image features are pre-extracted using ResNet-101 (He *et al.* 2016). Hyperparameter tuning for the Bayesian model is performed using the same grid search space as in Badirli *et al.* (2021).

## 5 Results

In this section, we describe our results on two evaluation tasks: DNA-specific tasks, designed to assess model performance in both open- and closed-world taxonomic settings; and zero-shot image classification using DNA embeddings.

### 5.1 DNA-specific categorization tasks

**Figure 3:**
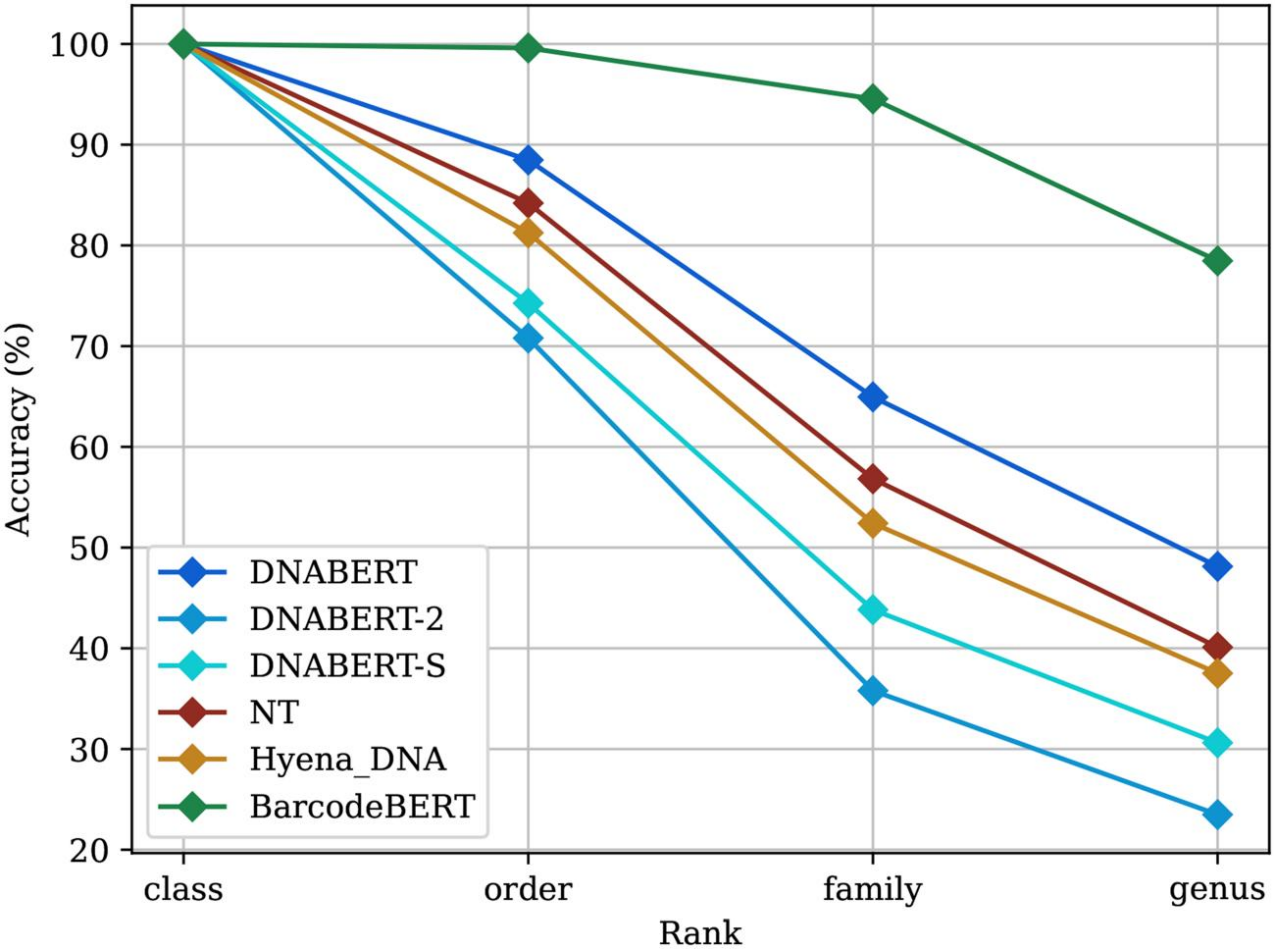
Comparison of different DNA foundation models on the task of 1-NN probing at different taxonomic levels. The query set contains DNA barcodes from species not present in the key set, and none of the models have undergone fine-tuning.

Our evaluation (Table 1) compares several models across species-level and genus-level DNA-specific categorization tasks (fine-tuning, linear probing, 1-NN probing, BIN reconstruction through ZSC). For species-level classification, a BLAST search yielded 99.7% accuracy based on the best hit. The performance of all fine-tuned deep learning-based models is comparable to this baseline, and all transformer models outperform the CNN model as well. DNABERT-2, DNABERT-S and BarcodeBERT all reached over 99.7% accuracy. Notably, only BarcodeBERT continues to closely match BLAST’s performance using a linear classifier, highlighting its strength in encoding meaningful features from raw data. In genus-level 1-NN probing, BarcodeBERT achieves the highest accuracy (78.5%) among the deep learning-based models—more than double that of the same architecture without pretraining—demonstrating that the pretrained model produces richer embeddings, making BarcodeBERT far more robust for similarity searches of sequences from novel taxa. BLAST, however, performs best in this task (83.9%). This indicates that without fine-tuning, BarcodeBERT captures coarser taxonomic distinctions but is limited in representing the full hierarchical taxonomic structure as shown in Fig. 3. In addition to accuracy, we computed the F1-score for each model across these tasks, and observed similar trends (see Supplement D.8, available as supplementary data at *Bioinformatics Advances* online).

**Table 1:** Classification accuracy of DNA barcode models under different SSL evaluation strategies and different efficiency metrics.^a^

			Species-level acc (%) of seen species			Genus-level 1-NN probe of unseen species		BIN reconstruction accuracy (%)
Model	#Param.	TPS (seq/s)	Finetuned	Linear probe	Dur (s)	Acc (%)	Dur (s)	ZSC probe
BLAST	N/A	N/A	***99.7*** ^b^		1495	**83.9**	602	N/A
CNN encoder	1.8 M	934	98.2	N/A	13	56.0	**55**	N/A
DNABERT (k=6)	88.1 M	50	99.5	98.1	248	48.1	1021	**81.5**
DNABERT-2	118.9 M	134	**99.7**	95.7	101	23.5	381	51.0
DNABERT-S	117.1 M	134	**99.7**	96.5	101	30.6	381	62.8
HyenaDNA-tiny (d256)	1.6 M	**1167**	99.4	93.5	**11**	50.6	76	52.8
Nucleotide Transformer	55.9 M	95	99.5	96.2	140	40.1	536	28.5
BarcodeBERT w/o pretraining	29.1 M	484	99.5	N/A	27	37.9	108	N/A
BarcodeBERT (4–4-4)	29.1 M	484	**99.7**	**99.2**	27	78.5	108	79.9

a The baselines are divided into three groups: alignment-based techniques, BLAST; a deep learning-based non-SSL CNN baseline; and off-the-shelf DNA foundation models pretrained on non-barcode data. These are compared against BarcodeBERT, which is specifically pretrained on DNA barcode-based datasets. For BarcodeBERT we used the best configuration of k=4, with four attention heads, and four layers. The DNABERT model supported variable stride length, and we show in parentheses the optimal *k*-mer value that yielded the best results. As an ablation, we also indicate the performance of the BarcodeBERT architecture when trained fully-supervised, without the self-supervised pretraining stage. Additionally, we show the throughput-per-second (TPS) of the encoders, and the total duration of the classification tasks. Numbers in boldface indicate the best result across each task, and underlined indicates second place. Note that alignment-free methods such as BWT and MESH are not comparable, as they are designed for longer genomic sequences (Langmead *et al.* 2009, Li and Durbin 2009, Ondov *et al.* 2016), making them unsuitable for short barcodes.

b BLAST is a deterministic algorithm without any learning component (see Supplement D.1, available as supplementary data at *Bioinformatics Advances* online, for details). Consequently, species classification accuracy does not correspond to fine-tuning or linear probing, and it is only included in the table for reference.

The ZSC task provides additional insights into the model’s understanding of the hierarchical taxonomic structure. High performance in ZSC alone indicates a learned representation’s ability to finely distinguish between closely related clusters (BINs) without necessarily capturing the higher-level taxonomy. In contrast, strong 1-NN performance at higher taxonomic levels but lower ZSC accuracy suggests that the model understands the overall topology of the hierarchical taxonomic structure, even if it lacks the granularity needed for precise clustering. DNABERT and BarcodeBERT exhibit this distinction, with BarcodeBERT achieving a more balanced performance across tasks, making it the more versatile model for comprehensive DNA barcode analysis.

Two efficiency measurements are included: throughput, defined exclusively for deep-learning-based models as the number of sequences processed per second, and total runtime for classification pipelines to ensure a fair comparison with alignment-based baselines. In terms of throughput, HyenaDNA(*tiny*) showcases the capabilities of state space models, demonstrating high throughput with fewer parameters. However, its classification performance is lower compared to BarcodeBERT and DNABERT-2. In total run time, our results indicate that subquadratic methods like the CNN baseline and HyenaDNA perform genus-level similarity searches (1-NN probe) 13× faster than BLAST, while BarcodeBERT is 5.6× faster than BLAST and outperforms other transformer models at this task. For species-level classification pipelines that do not include the computation of the training embeddings, transformer-based models demonstrate clear advantages over traditional baselines in terms of running time. Notably, BarcodeBERT, with a moderate parameter count, matches BLAST’s high classification accuracy (99.7%) with a 55× faster running time, thus providing a well-rounded option for large-scale DNA barcode applications. All efficiency experiments were conducted using an Intel(R) Xeon(R) CPU @ 2.20 GHz processor and a Quadro RTX 6000 GPU. Additional details on resource consumption can be found in Supplement C, available as supplementary data at *Bioinformatics Advances* online.

### 5.2 Zero-shot image classification using DNA embeddings

We use the Bayesian zero-shot learning task to evaluate the quality of the DNA feature embeddings, by assessing their effectiveness when used as side information for classifying images to species on the INSECT dataset. We consider the embeddings directly from the pretrained models and also after fine-tuning. The accuracy for seen and unseen test species and the harmonic mean are presented in Table 2. Even without fine-tuning, BarcodeBERT substantially outperforms DNABERT and DNABERT-2 on unseen species, regardless of whether they had been fine-tuned previously. BarcodeBERT achieves similar performance to the reported baseline CNN results (Badirli *et al.* 2021) and improves on the harmonic mean score by 1.2% and unseen accuracy by 1.9%, respectively. Our results demonstrate that in the zero-shot learning task of predicting insect species, employing BERT-like models that have also been trained on insect DNA barcodes as DNA encoders can improve performance over CNNs and general DNA foundation models.

**Table 2:** Evaluation of DNA barcode models in a Bayesian zero-shot learning task on the INSECT dataset.^a^

	Data sources		Species-level acc (%)		
Model	SSL pretraining	Fine-tuning	Seen	Unseen	Harmonic Mean
CNN encoder	–	Insect	38.3/39.4	**20.8**/18.9	**27.0**/**25.5**
DNABERT	Human	–	35.0	10.3	16.0
DNABERT	Human	Insect	**39.8**	10.4	16.5
DNABERT-2	Multi-species	–	36.2	10.4	16.2
DNABERT-2	Multi-species	Insect	30.8	8.6	13.4
BarcodeBERT (ours)	Invertebrates	–	31.6	**20.0**	24.5
BarcodeBERT (ours)	Invertebrates	Insect	38.8	15.3	22.0

a The pretraining and fine-tuning data source is indicated by the respective DNA type, and ‘–’ signifies the absence of training for that type. We also indicate the most specific taxon subset. For the baseline CNN encoder, we report the original paper result (left) and our reproduction (right). Numbers in boldface indicate the best result across each task, and underlined second best.

### 5.3 Ablation studies

In our main results, we demonstrated the utility of self-supervised pretraining BarcodeBERT to enable our model to generalize to open-world tasks. In this section, we study the impact of the different components involved in pretraining. We use the terminology context tokens for tokens that are left unchanged to provide context to the model during pretraining and the terminology substitution tokens for tokens that will be changed for the masked language modeling task. We consider different strategies to calculate the loss of each group separately. The loss associated with predicting contextual tokens is referred to as the “context component” of the loss (although predictions in the foundation model literature are typically restricted to substitution tokens, we extend this to include context token predictions, maintaining the terminology to explore their utility in input reconstruction similar to autoencoders), while the loss related to predicting substitution tokens is the “substitution component.” By assigning different weights to these two loss components, we sought to observe how these adjustments would affect both training and evaluation. In particular, we define $w_{s}$ as the penalty weight given to the substitution component of the loss. Note that $1-w_{s}$ is always the weight of the context component of the loss.

### 5.4 Substitution token rate

To examine how varying the substitution token ratio ($r_{s}$) affects performance, we tested several ratios, keeping the model architecture (four attention heads, four layers), tokenization (k=4), and substitution penalty weight ($w_{s}=1$) constant. Table 3 shows that species-level classification performance remains consistently high across substitution rates, peaking at 99.67% accuracy with 45% and 50% substitution tokens. Linear probe results align closely, reaching the highest accuracy of 99.02% at the 50% substitution rate. For genus-level 1-NN probing of unseen species, the 50% substitution rate yields the best accuracy at 78.47%, suggesting that this rate provides a balance that strengthens the model’s ability to generalize to new taxa. Lower substitution rates show slightly reduced generalization, while a 60% rate begins to degrade performance, indicating that 50% is the optimal value for $r_{s}$.

**Table 3:** Classification accuracy over the different substitution token ratios $r_{s}$, while keeping constant all of the model architecture (4–4), the value of k=4 during tokenization and the penalty weight for the substitution component of the loss ($w_{s}=1$).^a^

Substitution token ratio (%)	Species-level acc (%) of seen species		Genus-level acc (%) of unseen species
	Fine-tuned	Linear probe	1-NN probe
15	98.95	98.95	75.15
30	98.79	98.79	74.24
45	**99.67**	98.54	74.42
→ *50* ←	**99.67**	**99.02**	**78.47**
60	99.62	98.45	77.56

a Numbers in boldface indicate the highest accuracies and the italic value inside → selected ← shows the selected optimal parameter.

### 5.5 Weight of the substitution component of the loss

Building on the fact that predicting context tokens is inherently easier than predicting substitution tokens for LLMs, we investigated how adjusting the penalty weights between these two tasks affects the performance of the model. For this purpose, we experimented with different penalty weights assigned to the substitution component of the loss ($w_{s}$). Table 4 provides the accuracy for genus-level 1-NN probing of unseen species across different values for $w_{s}$ in combination with four *k*-mer sizes (2, 4, 6, and 8) and Byte-Pair Encoding (BPE) tokenizer obtained from DNABERT-2. Alternative BPE tokenizers that specifically fit our data are investigated later in this section. We kept the architecture (four layers, four attention heads) and substitution token rate ($r_{s}=50\%$) constant. As shown in Table 4, the optimal performance across all *k*-mer sizes was achieved with a $w_{s}$ of 1.0, where the highest accuracy, 78.47%, was observed with k=4.

**Table 4:** Genus-level accuracy for 1-NN probing of unseen species with varying penalty weight assigned to the substitution component of the loss ($w_{s}$).^a^

	Genus-level acc (%) of				
	unseen species with 1-NN probe				
Loss weight ($w_{s}$)	k=2	k=4	k=6	k=8	BPE
0.2	64.18	76.06	75.15	71.15	70.57
0.5	66.47	74.98	76.62	71.22	70.34
0.8	68.84	76.71	74.66	73.33	69.40
0.9	69.51	77.16	76.06	72.23	67.48
→ *1.0* ←	76.92	**78.47**	75.74	75.62	69.85

a The model architecture remains fixed (4–4), with substitution token ratio $r_{s}$ = 50%. Two tokenizers were tested: a *k*-mer tokenizer with *k*-mer sizes of 2, 4, 6, and 8, and a BPE tokenizer used in DNABERT-2 with a fixed vocabulary size of 4096. Note that the weight of the context component of the loss always equals $1-w_{s}$. The number in boldface indicates the overall best accuracy, underlined the best per tokenizer, and italic the → selected ← optimal parameter.

Our experiments indicate that focusing the loss penalty on the harder task of predicting substitution tokens, while not penalizing the easier task of predicting context tokens, yields the best accuracy. This aligns with observations in other foundation models, such as BERT and DNABERT (see Supplement C, available as supplementary data at *Bioinformatics Advances* online, for more details).

### 5.6 Tokenization strategies

We evaluated the BPE tokenizer, which generates variable-length tokens based on character co-occurrence frequencies (Sennrich *et al.* 2016). BPE was designed for subword tokenization to overcome fixed vocabulary (Sennrich *et al.* 2016) by compressing sequences for efficient representation (Gallé 2019). Unlike overlapping *k*-mers, BPE has an advantage for masked DNA language models (Zhou *et al.* 2023) as it avoids information leaks from adjacent masked tokens. We used DNABERT-2’s BPE tokenizer, trained on 2.75 billion nucleotide bases from the human nuclear genome and 32.49 billion bases from 135 species across various kingdoms. We also trained custom BPE tokenizers with varying vocabulary sizes on our DNA barcode dataset and evaluated across various training scenarios on the genus-level 1-NN probing task. Based on results in Table 4, we selected a loss weight of 1.0 for best performance. Our setup used a 50% substitution token ratio ($r_{s}$). Full results across configurations are shown in Table 5.

**Table 5:** Genus-level accuracy for unseen species with different tokenizers, various model sizes, fixed weight for the substitution component of the loss function ($w_{s}=1$) and a substitution token ratio $r_{s}=50\%$.^a^

	*k*-mer tokenizer				DNABERT-2 BPE	BarcodeBERT BPE			
Model size	k=2	k=4	k=6	k=8	v=4096	v=4096	v=1024	v=256	v=128
4 Layers, 4 heads	76.92	**78.47**	75.74	75.62	69.85	66.88	68.58	66.57	63.42
6 Layers, 6 heads	71.46	**76.95**	76.04	76.60	70.17	67.30	66.95	63.49	60.61
12 Layers, 12 heads	74.71	70.17	70.80	**75.81**	68.68	67.79	62.39	56.94	54.09

a Two types of tokenizers were tested: a *k*-mer tokenizer with *k*-mer sizes of 2, 4, 6, and 8, and five different versions of BPE tokenizers. BPE tokenizer from DNABERT-2 has a fixed vocabulary size of 4096, while BPE tokenizers trained on our dataset have vocabulary sizes (*v*) of 4096, 1024, 256, and 128. Numbers in boldface indicate the best result across each architecture.

#### 5.6.1 Model size

We report genus-level 1-NN probing accuracy on the unseen data for DNABERT-2 BPE and BarcodeBERT BPE tokenizers across three model sizes. Models using DNABERT-2’s BPE showed no significant accuracy changes across different sizes. However, for BarcodeBERT BPE, we see that increasing the model size reduces the accuracy across different vocabulary sizes, possibly due to overfitting. For additional experiments, see Supplementary D.3, available as supplementary data at *Bioinformatics Advances* online.

#### 5.6.2 Vocabulary size

We trained new BPE tokenizers with varying vocabulary sizes (*v*) to evaluate their impact on BarcodeBERT BPE performance. Unlike *k*-mer tokenizers, BPE sequence lengths vary with input composition and require padding or truncation to a maximum length before tokenization. Since smaller vocabularies (*v*) produce longer sequences (see Supplement B.1, available as supplementary data at *Bioinformatics Advances* online), we set maximum lengths of 128 for v∈{4096,1024} and 256 for v∈{256,128}. Reducing the vocabulary size from 4096 to 128 consistently decreases accuracy across all model sizes.

#### 5.6.3 Comparing k-mer with BPE

We find that *k*-mer tokenizers outperform BPE tokenizers in all model configurations. We hypothesize that this is likely due to three reasons. First, DNA barcode sequences are too short to benefit from the BPE compression. Second, BPE is sensitive to minor variations such as single-character substitutions, which is unsuitable for DNA datasets with single-nucleotide mutations in arbitrary positions. In other words, BPE tokenization varies greatly for similar sequences with small Hamming distances, while *k*-mer tokenization remains consistent, as a single-nucleotide substitution affects only one token (see Fig. 9, available as supplementary data at *Bioinformatics Advances* online). Third, although BPE handles small sequence alignment shifts better than *k*-mer tokenizers, this can be mitigated in *k*-mers with data augmentation using random offsets during pretraining (see Table 6, available as supplementary data at *Bioinformatics Advances* online).

## 6 Discussion

This study presents the first application of deep learning to an extensive DNA barcode collection of Canadian invertebrates. By encoding each DNA barcode into a fixed-length embedding, BarcodeBERT achieves strong scalability, with computation time remaining constant even as reference databases such as BOLD (growing by 3–4 million records per year) continue to expand. BarcodeBERT is also more versatile than traditional approaches, supporting diverse downstream applications, including multimodal models that integrate DNA with image data (Gong *et al.* 2025), thus extending beyond the capabilities of alignment-based approaches like BLAST.

Our results demonstrate that pretraining masked language models on DNA barcode data, as exemplified by BarcodeBERT, is highly effective for arthropod species identification. Despite only 35% of the pretraining data being labeled at the species rank, the model learns latent features that purely supervised approaches cannot capture. BarcodeBERT performs well on key biodiversity tasks, such as taxonomic classification, clustering, and similarity searches, benefiting from efficient hardware acceleration that enables scaling for large datasets and achieving 55× faster species-level classification than alignment-based methods. By systematically evaluating tokenization and masking strategies, this study also provides guidance for the pretraining of DNA-specific foundation models.

Despite its strengths, BarcodeBERT has some limitations. Its training data may have taxonomic and geographical biases as the model is trained exclusively on invertebrate species from Canada, potentially limiting its applicability in global studies. The BOLD dataset, comprising more than 16 million DNA barcodes from a wide geographical distribution (Ratnasingham and Hebert 2007), represents untapped data that could address such biases. Thus, future work should incorporate more diverse datasets to develop robust, globally scalable models for taxonomic classification. Additionally, while longer genomic sequences could offer deeper insights for specialized phylogenetic analyses, the quadratic time complexity of transformer models limits their applicability to such sequences. Future work could include more compute-efficient architectures, such as structured state space models, which scale sub-quadratically with sequence length (Gu *et al.* 2022).

We also note several other directions for further research. Our methodology and findings could be broadly applicable to taxonomic classification of barcode regions of species from other kingdoms, such as the ITS region for fungi (Schoch *et al.* 2012). Architecturally, BarcodeBERT could be extended, e.g. with multiple classification heads to jointly predict other taxonomic ranks such as phylum, class, order, or genus. Lastly, the non-parametric classification approach using learned embeddings for similarity searches could potentially be extended to handle sequences from novel taxonomic ranks and make predictions at higher taxonomic levels.

## 7 Conclusions

BarcodeBERT leverages 1 million DNA barcodes with partial taxonomic annotations to outperform state-of-the-art foundation models in genus-level and species-level classification tasks. Notably, BarcodeBERT matches the high accuracy of the alignment-based classification tool BLAST in species classification, while being 55× faster and more scalable. In addition, our extensive analysis of pretraining strategies provides practical guidance for building customized DNA language models for large-scale taxonomic classification.

Overall, BarcodeBERT’s performance demonstrates how transformer-based architectures can be successfully customized to overcome the challenges of genomic biodiversity data for effective DNA barcode identification and classification. Lastly, not being limited to a specific dataset or barcode region, our model is highly amenable to future applications, to global datasets or barcode regions in other kingdoms of life.

## Supplementary Material

vbag054_Supplementary_Data
