# Supplementary material for: BarcodeBERT: transformers for biodiversity analyses
Source: Bioinform Adv. 2026 Feb 19;6(1):vbag054. doi: 10.1093/bioadv/vbag054 (PMC13008329; doi:10.1093/bioadv/vbag054)
Supplement: vbag054_Supplementary_Data [file vbag054_supplementary_data.pdf]

Supplementary Materials for the paper “BarcodeBERT: Transformers for biodiversity analyses”

Pablo Millan Arias, Niousha Sadjadi, Monireh Safari,
ZeMing Gong, Austin T. Wang, Joakim Bruslund Haurum, Iuliia Zarubiieva,
Dirk Steinke, Lila Kari, Angel X. Chan, Scott C. Lowe, and Graham W. Taylor

A Dataset Description

After pre-processing (see *"Data pre-processing"* in the main text), the final dataset contained 965,289 samples. The dataset was divided into three main partitions: *Pretrain*, *Seen*, and *Unseen*. The *Seen* partition was subdivided into supervised training, validation, and testing subsets. Each partition serves distinct experimental purposes, with the *Unseen* partition designed to mimic real-world scenarios where models encounter sequences from previously unobserved species. Our preprocessed version of the dataset is available to download at `https://huggingface.co/datasets/bioscan-ml/CanadianInvertebrates-ML`.

Table S1 summarizes the composition of each partition, detailing the number of unique records across taxonomic categories, from phylum to species. It also includes the number of unique Barcode Index Numbers (BINs); each BIN serves as a species-level proxy, created by clustering similar DNA barcode sequences into a molecular taxonomic unit. The *Pretrain* partition contains 893,744 sequences from 15 phyla, while the *Seen* and *Unseen* partitions are more specific, containing sequences primarily from phylum Arthropoda. The *Unseen* partition contains 4,278 barcodes from 1,826 species absent from the training and validation subsets.

Table S1: Number of unique records across taxonomic categories in each partition.

Partition	Phylum	Class	Order	Family	Genus	Species	DNA barcode	BIN
Pretrain	15	49	176	1,188	6,679	14,794	893,744	62,489
Test	1	1	13	161	500	1,653	13,460	2,125
Train	1	1	13	161	500	1,653	47,086	2,430
Validation	1	1	13	161	500	1,653	6,721	1,954
Unseen	1	1	13	161	500	1,826	4,278	1,885

To verify the overlap between partitions, Tables S2 and S3 provide pairwise comparisons of shared species and genera, respectively. As expected, there is no overlap in species between the *Unseen* partition and other subsets, ensuring the appropriateness of the *Unseen* partition for evaluating generalization. That being said, some genera are shared across partitions, reflecting the hierarchical taxonomic structure of the data. All the subsets in the *Seen* partition share all 500 genera that are also present in the *Unseen* partition. The *Pretrain* partition shares 467 of these genera with each of the others.

Table S2: Pairwise comparison of species overlap between partitions, with each cell representing the number of species shared between two partitions. The total number of unique species within a partition is given along the diagonal.

	Pretrain	Unseen	Train	Test	Validation
Pretrain	14,794	0	809	809	809
Unseen	0	1826	0	0	0
Train	809	0	1653	1653	1653
Test	809	0	1653	1653	1653
Validation	809	0	1653	1653	1653

Table S3: Pairwise comparison of genus overlap between partitions, with each cell representing the number of genera shared between two partitions. The total number of unique genera within a partition is given along the diagonal.

	Pretrain	Unseen	Train	Test	Validation
Pretrain	6679	467	467	467	467
Unseen	467	500	500	500	500
Train	467	500	500	500	500
Test	467	500	500	500	500
Validation	467	500	500	500	500

Table S4 details the number of unique records across taxonomic ranks for each phylum in the *Pretrain* partition. Arthropoda dominates this partition, accounting for over 95% of the sequences, followed by smaller contributions from other phyla like Mollusca and Annelida. Table S5 highlights the percentage of labelled records for each phylum, showing significant gaps in taxonomic annotations at the species and genus levels, particularly for Arthropoda, where less than 40% of the sequences are labelled at the species level.

Table S4: The distribution of barcode sequences across taxonomic ranks for each phylum used in the *Pretrain* partition.

Phylum	Class	Order	Family	Genus	Species	BIN
Annelida	2	16	48	150	329	516
Arthropoda	14	67	929	6,211	13,991	61,328
Brachiopoda	1	2	2	2	2	2
Bryozoa	3	3	3	2	2	4
Chordata	5	18	37	67	89	102
Cnidaria	4	10	24	25	24	46
Echinodermata	5	17	26	43	74	79
Hemichordata	1	1	1	2	1	2
Mollusca	6	30	97	162	271	372
Nematoda	2	5	10	5	2	8
Nemertea	3	2	5	5	5	22
Platyhelminthes	0	0	0	0	0	1
Porifera	1	3	4	4	3	5
Priapulida	1	1	1	1	1	1
Tardigrada	1	1	1	0	0	1

Figure S1 illustrates the species frequency across partitions, sorted in decreasing order. The subsets in the *Seen* partition exhibit a relatively uniform distribution, while the *Unseen* partition contains a higher proportion of rare species. Figures S2 and S3 present sunburst plots for the supervised test subset and the *Unseen* partition, visualizing the taxonomic hierarchy from order to genus. These figures highlight the differences in taxonomic composition between partitions, with the supervised test subset showing a more balanced distribution compared to the *Unseen* partition, which emphasizes underrepresented genera and families.

Lastly, we analyzed the variability of COI DNA barcodes across insect species in the *test* partition using the normalized edit distance and cosine distance on the learned em-

Table S5: Percentage of labelled records across taxonomic ranks for each phylum in the *Pretrain* partition.

Phylum	Class	Order	Family	Genus	Species	BIN
Annelida	100	96.43	99.81	87.44	80.35	97.95
Arthropoda	100	100	99.97	66.91	35.05	99.37
Brachiopoda	100	100	100	100	90.00	90.00
Bryozoa	80.00	80.00	80.00	40.00	40.00	100
Chordata	100	99.65	98.62	98.27	94.81	96.89
Cnidaria	100	98.21	95.54	84.82	54.46	99.11
Echinodermata	100	100	100	100	100	100
Hemichordata	100	100	100	100	25.00	100
Mollusca	99.95	89.02	99.69	98.43	90.22	96.71
Nematoda	100	100	91.67	54.17	16.67	37.50
Nemertea	96.43	94.64	87.50	64.29	48.21	100
Platyhelminthes	0	0	0	0	0	100
Porifera	100	100	100	85.71	42.86	85.71
Priapulida	100	100	100	100	100	100
Tardigrada	100	100	100	0	0	100

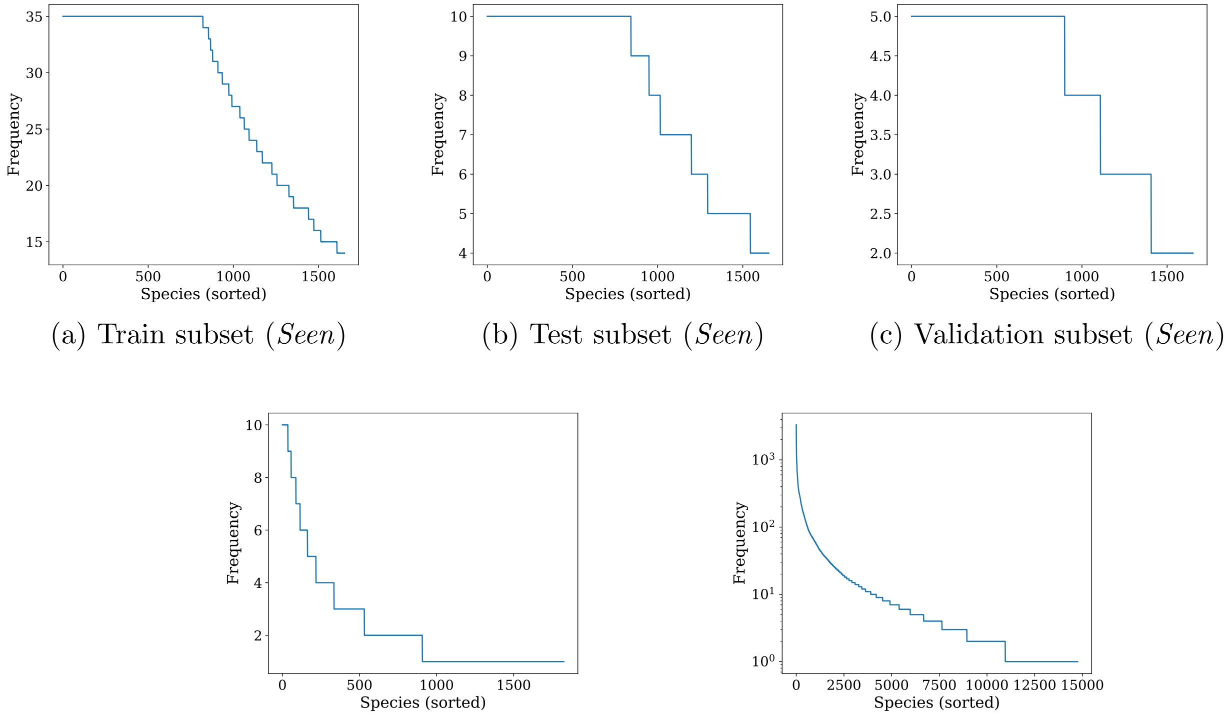

(a) Train subset (*Seen*) (b) Test subset (*Seen*) (c) Validation subset (*Seen*)

(d) *Unseen* partition (e) *Pretrain* partition

Figure S1: Frequency of species records across each partition, sorted in decreasing order.

beddings. Only species with more than two sequences are considered, and for each species $\mathcal{S}$, we computed both intra- and inter-species distances. Specifically, for each sequence, we calculated:

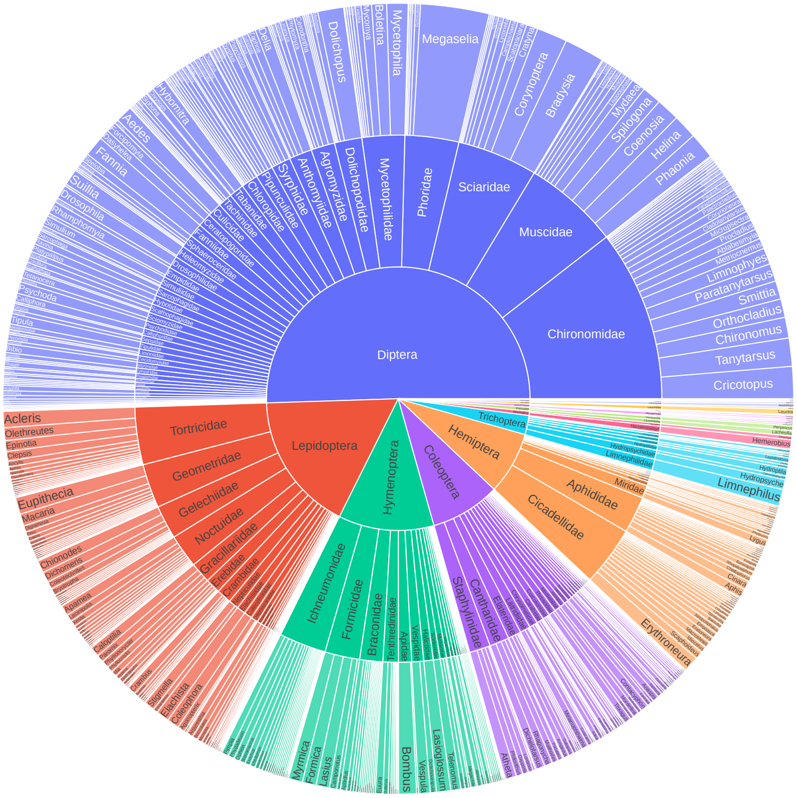

Figure S2: Sunburst plot of taxonomic distribution in the test subset of the *Seen* partition, with each radial level representing a taxonomic rank from order to genus. An interactive version of this plot is available at Test_Seen.html

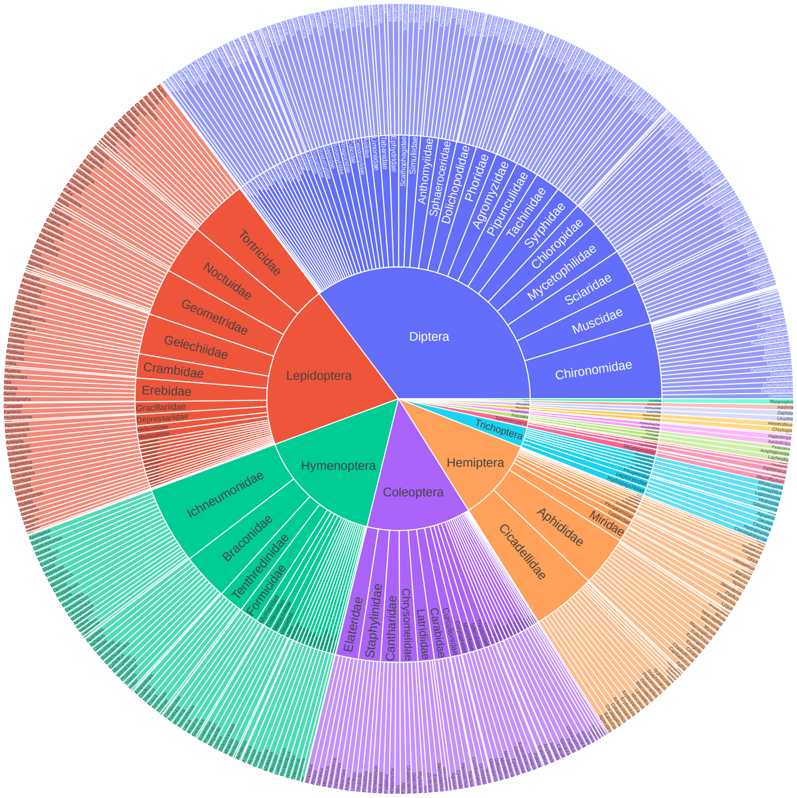

Figure S3: Sunburst plot of taxonomic distribution in the *Unseen* partition, with each radial level representing a taxonomic rank from order to genus. An interactive version of this plot is available at Unseen.html

.

- The distance to all other sequences within the same species (intra-species), and
- The distance to all sequences from different species (inter-species),

while ensuring distances were not double-counted. Finally, we estimated each distribution via Gaussian kernel density estimation (KDE) and computed overlap by integrating the minimum of the two KDE curves.

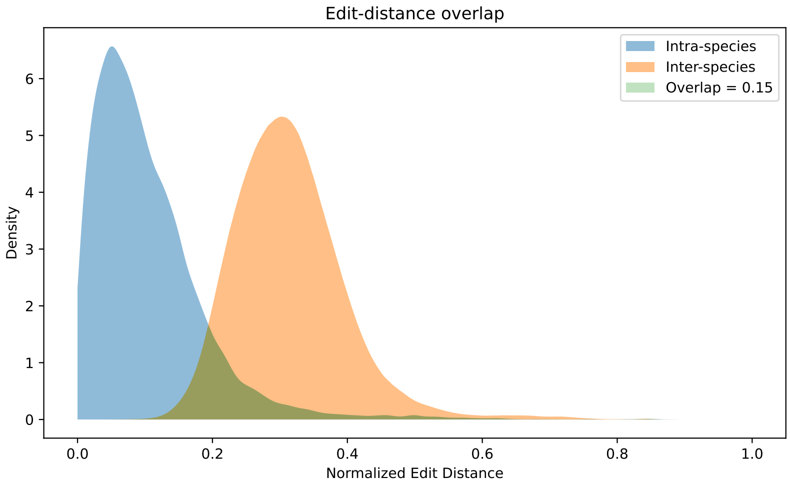

Figure S4: Distribution of intra- and inter-species distances based on **normalized edit distance** computed on the test partition. **Blue** represents intra-species distances, **orange** represents inter-species distances, and the area of overlap is shaded in **green**. Distances were calculated using Levenshtein alignment, normalized using min-max normalization. Species with at least two sequences were included in the analysis.

The edit distance (Figure S4) provides a clear separation between intra- and inter-species distributions, with minimal overlap (0.15). These distributions quantitatively capture the natural variability in COI sequences and illustrate the potential for learning taxonomic structure with deep models. This intuition is confirmed by the results obtained after estimating the distribution of the cosine distances of the learned embeddings (Figure S5). Indeed, self-supervised pretraining enables BarcodeBERT to encode biologically relevant distinctions more clearly than raw alignment metrics with an overlap of 0.02 between both distributions. While these distances reflect biological structure and are useful in our k-NN classifier and zero-shot classification (ZSC) settings, the separation between intra- and inter-species groups should be further investigated, especially constrained to other taxonomic ranks.

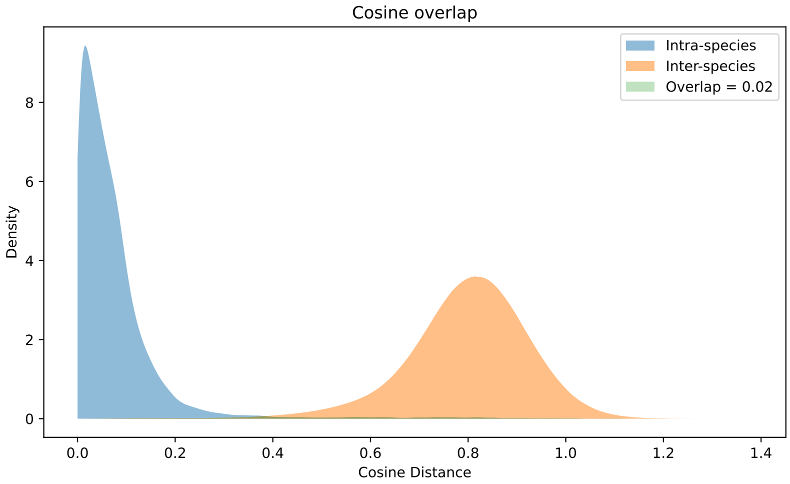

Figure S5: Distribution of intra- and inter-species distances based on cosine distance computed on GAP embeddings generated by the model. **Blue** represents intra-species distances, **orange** represents inter-species distances, and the area of overlap is shaded in **green**. While cosine distances on learned embeddings capture biological structure, the separation between intra- and inter-species groups is less distinct than with alignment-based metrics.

B Tokenization Strategies — Extra results

B.1 Variation in Length of Tokenized Sequences

Compared to the k-mer tokenizer, which generates a tokenized sequence proportional to the DNA sequence length, the length of the tokenized sequence produced by the BPE tokenizer is not directly determined by the nucleotide sequence length and can vary depending on the composition of the input sequence and the vocabulary size of the BPE tokenizer.

Figure S6 shows the distribution of tokenized sequence lengths in DNA barcode pretraining data. Generally, BPE tokenizers with smaller vocabulary sizes tend to produce longer tokenized sequences, with the only exception being the DNABERT-2 BPE. According to Figure S6, although the tokenizer trained on DNABERT-2 uses a vocabulary size of 4096, it generates relatively long tokenized sequences. This is because the dataset used to train DNABERT-2 differs from the DNA barcode dataset used to create other tokenizers.

B.2 Sensitivity of Tokenizers to DNA Sequence Variations

There are two main ways to measure similarity between sequences. One is through the Hamming distance, which counts the number of mismatches between two strings, i.e., the number of positions where the strings do not share the same nucleotide. The other is the edit distance, also called the Levenshtein distance, which is defined as the minimum number of string operations (substitutions, insertions, deletions) required to transform one string into the other. In comparative genomics, the edit distance is widely used to estimate evolutionary relationships, as it corresponds to a better model of true sequence similarity. However,

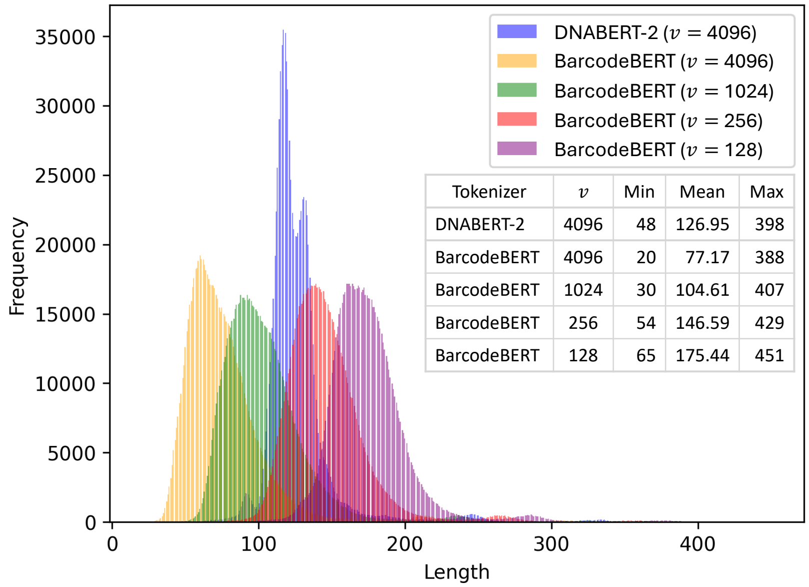

Tokenizer	v	Min	Mean	Max
DNABERT-2	4096	48	126.95	398
BarcodeBERT	4096	20	77.17	388
BarcodeBERT	1024	30	104.61	407
BarcodeBERT	256	54	146.59	429
BarcodeBERT	128	65	175.44	451

Figure S6: Distribution of tokenized sequence lengths across different BPE tokenizers, shown as histograms for the DNA barcode pretraining data. The distributions correspond to different BPE tokenizers, including DNABERT-2 and BarcodeBERT with vocabulary sizes (v) of 4096, 1024, 256, and 128. Smaller vocabulary sizes result in longer tokenized sequences, as reflected by the rightward shift in the distribution. Summary statistics (minimum, mean, and maximum lengths) are provided in the table inset.

the Hamming distance can be calculated in linear time, even in token space with larger vocabularies. Figure S7 illustrates how the Hamming distance varies with a single insertion operation versus a single substitution operation in both sequence space and token space for a pair of sequences. We include the two tokenization strategies considered in our study: k-mer and DNABERT-2 BPE tokenization. In both scenarios, the edit distance is equal to one, but just the BPE tokenizer can model this behaviour using the Hamming distance as a proxy.

To better understand the relationships between string distances and their practical utility, we considered 1000 different genera in the *Pretraining* partition and sampled a pair of sequences from two different species within each genus. Figure S8 illustrates how the edit distance varies with the Hamming distance in the experiment. Two salient regions are evident in the plot. In the “aligned region”, the edit distance closely follows the Hamming distance as the number of mismatches is not due to alignment issues. In contrast, the “non-aligned region” potentially involves scenarios where sequences have been shifted, rearranged, or otherwise misaligned, leading to increased apparent mismatches when aligned positionally. These mismatches can be resolved with fewer string operations, resulting in a considerably lower edit distance compared to the Hamming distance.

The Hamming distance in the token space of an optimal tokenizer should closely follow the behaviour of the edit distance in both regions. This is an indicator of how much the tokenizer is helping in modelling the true sequence similarity. To study the behaviour of

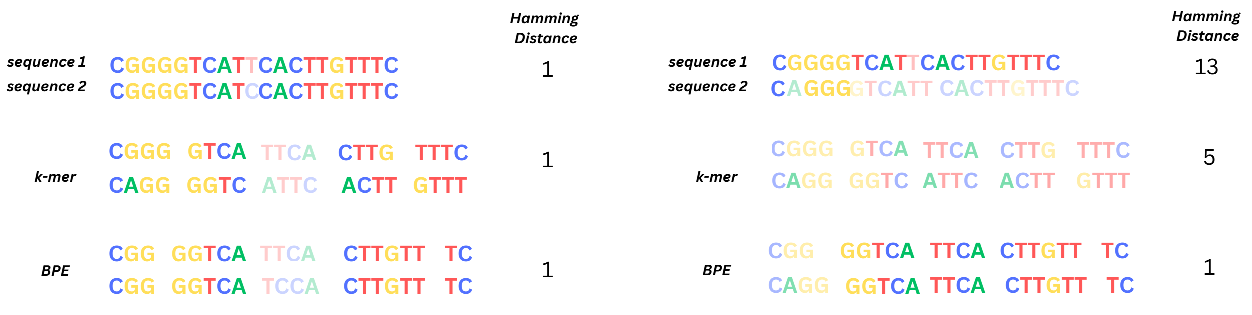

(a) Substitution (b) Insertion

Figure S7: Illustration of the differences between the Hamming distances in sequence and token space using k-mer and DNABERT-2 BPE tokenization strategies for two different string operations where the edit distance between a pair of sequences is equal to one.

both tokenization strategies, we repeated the experiment with 1000 random genera from the pretraining dataset and visualized a scatter plot of the Hamming distance in token space versus the Hamming distance in sequence space for these sequences for both BPE and k-mer tokenizers.

As illustrated in Figure S9, in the “aligned region”, the k-mer tokenizer closely corresponds to the Hamming distance scaled by a factor of $1/k$. However, the BPE tokenizer is sensitive to minor changes in the sequences, possibly due to substitutions that lead to unknown tokens, causing the tokenizer to split these unknown tokens into single nucleotides. Even when the Hamming distance between two DNA sequences is small, their tokenized representations can differ significantly with BPE tokenization compared to k-mer tokenization.

On the other hand, the BPE tokenizer is more robust to frame shifts, following the behaviour of the edit distance in the “non-aligned region” of the plot. The k-mer tokenizer struggles in this region, as large Hamming distances can sometimes result from small frame shifts in DNA sequences, which is also illustrated in Figure S7. This limitation can be addressed by incorporating data augmentation during the pretraining phase to increase the model’s robustness to frame shifts by randomly offsetting the input sequence.

Table S6 shows the effect of pretraining with a random offset augmentation on the downstream genus-level accuracy for unseen species when using k-mer versus BPE tokenization with the BarcodeBERT model (4 layers, 4 attention heads). The k-mer length is set to $k = 4$, and for both tokenizers the offset is randomly selected between 0 and 3 (inclusive). We use a weight of 1 for the substitution component of the loss function ($w_s = 1$), a substitution token ratio of 50% ($r_s = 50\%$), and the proportion of substitution tokens assigned to `[MASK]` set to 1 ($r_{\texttt{[MASK]}} = 1$). As shown in Table S6, random offset augmentation improves the genus-level accuracy of the k-mer-based model by 4.27 percentage points, but marginally *decreases* the performance of the model trained with the BPE tokenizer.

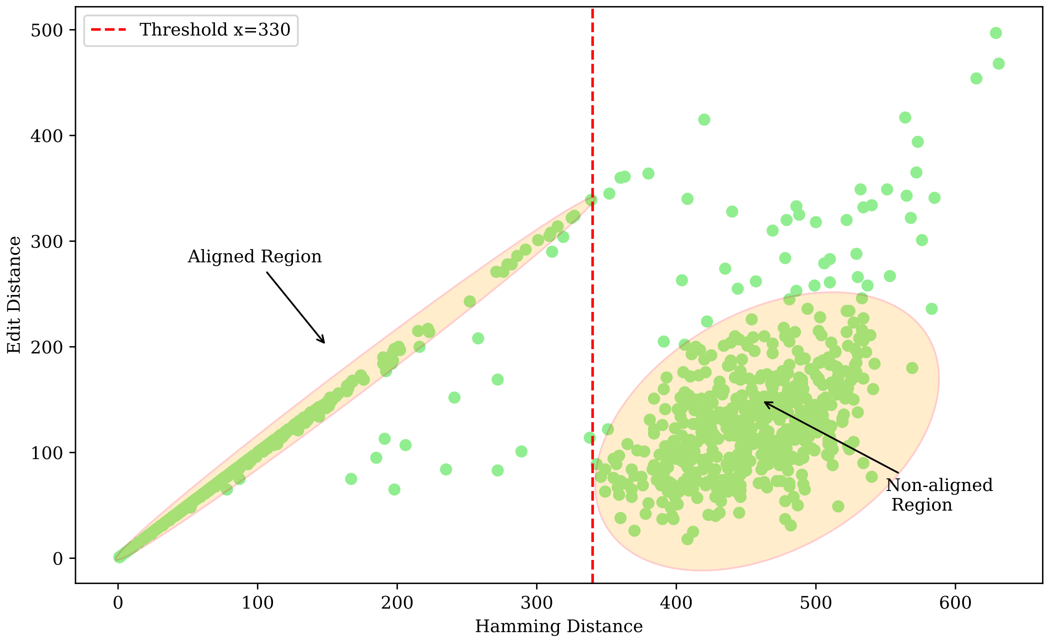

Figure S8: Relationship between edit distance and Hamming distance for pairs of sequences sampled from different species across 1000 genera. The plot highlights two regions: the "aligned region", where the edit distance and Hamming distance are closely matched, and the "non-aligned" region, where misalignments result in greater Hamming distances relative to the edit distance.

Table S6: Effect of augmenting with random offsets to the DNA sequences before tokenization with either k-mer or BPE tokenizers. In all experiments, we used a BarcodeBERT model with 4 layers and 4 attention heads, fixed weight for the substitution component of the loss function ($w_s = 1$), substitution token ratio ($r_s = 50\%$) and substitution token proportion assigned to `[MASK]`, ($r_{\texttt{[MASK]}} = 1$).

Tokenizer	Random Offset	Genus-level acc (%) of unseen species with 1-NN probe
k-mer	✗	74.20
	✓	78.47 ↑4.27
BPE	✗	69.85
	✓	68.56 ↓1.29

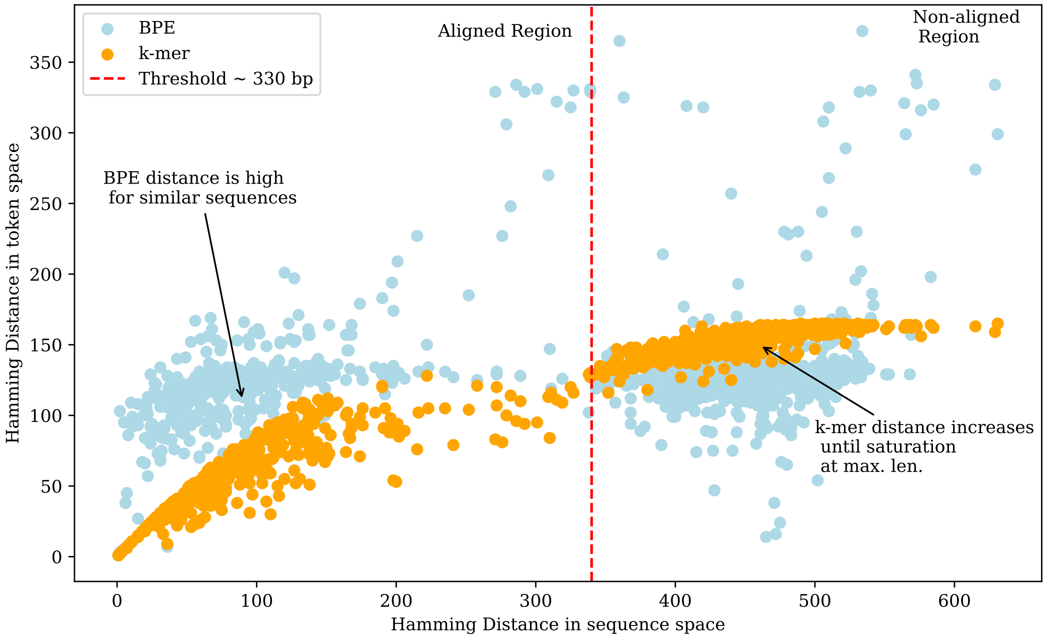

Figure S9: Comparison of Hamming distances in token space versus sequence space for pairs of sequences sampled from different species across 1000 genera. For both tokenizers, each dot in the plot represents the distances between sequences in a specific genus.

C Masking Strategy

In this supplementary experiment, we adapted our masking strategy to mimic BERT's original methodology [2]. BERT addresses the difference in token distribution between pretraining and fine-tuning by employing a masking strategy whereby 80% of substitution tokens are replaced by `[MASK]` tokens, 10% are replaced with random tokens, and 10% remain unchanged. This approach ensures more robust embeddings during testing, where masked tokens are absent [2].

To incorporate this methodology into BarcodeBERT, we define the following terms: $r_{\texttt{[MASK]}}$ as the proportion of substitution tokens assigned to the `[MASK]` token and $r_{\texttt{[RAND]}} = 1 - r_{\texttt{[MASK]}}$ as the proportion of the substitution tokens assigned a random valid token (all tokens except the special tokens). We explored various ratios for token replacement and three different values for the substitution loss penalty w_s. In the first case, based on the results of our experiments, where $w_s = 1$ had the best performance, we kept $w_s = 1$ and adjusted $r_{\texttt{[MASK]}}$. In the second case, we set w_s to 0.95 and in the third case, w_s was set to 0.90, to closely replicate BERT's original strategy that keeps 10% of the tokens unchanged. Note that in all experiments $r_{\texttt{[RAND]}}$ was set to $1 - r_{\texttt{[MASK]}}$. In this study, we used the best configuration of 4 layers and 4 attention heads, $k = 4$, and $r_s = 50\%$. Figure S10 presents the accuracy of these experiments for genus-level 1-NN probing on unseen species. The results show that for $w_s = 1.0$, the best accuracy is 78.47% with $r_{\texttt{[MASK]}} = 1.0$, for $w_s = 0.95$ the best accuracy is 76.85% with $r_{\texttt{[MASK]}} = 0.9$, and for $w_s = 0.9$, $r_{\texttt{[MASK]}} = 0.5$ gives the best accuracy of 78.14%, which improves the accuracy by 1% compared to the case where $w_s = 0.9$ and $r_{\texttt{[MASK]}} = 1.0$. Our results demonstrate that adopting BERT's masking strategy did not enhance the performance of BarcodeBERT, indicating that maintaining $r_{\texttt{[MASK]}} = 1$ is the optimal configuration.

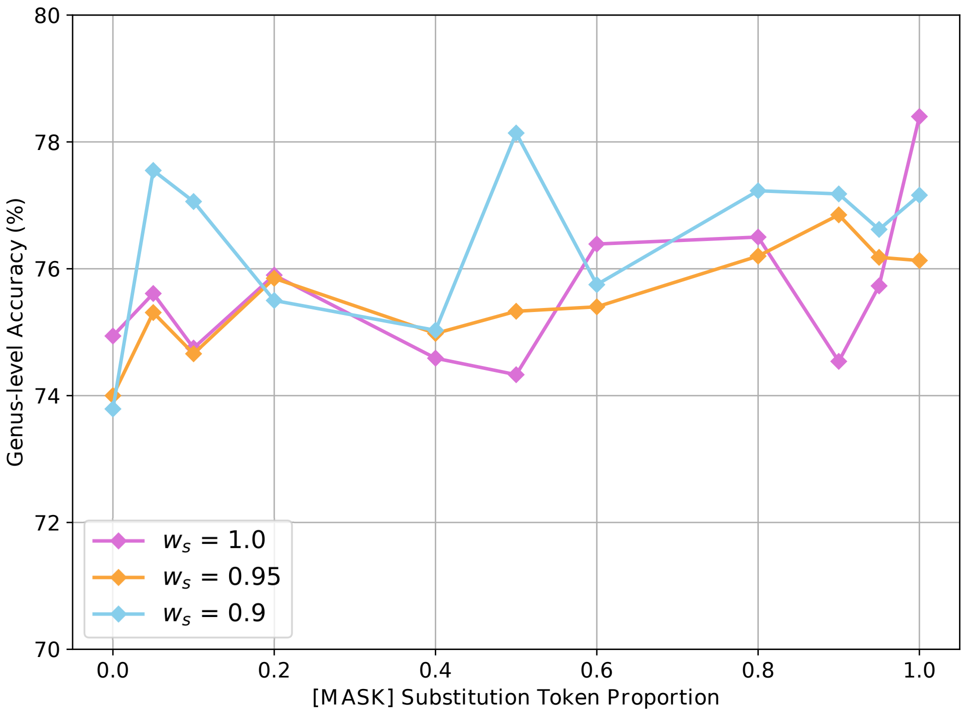

Figure S10: Genus-level accuracy for 1-NN probing of unseen species across different values of $r_{\texttt{[MASK]}}$, $r_{\texttt{[RNAD]}}$, and w_s. Experiments were conducted using the optimal configuration: 4 layers, 4 attention heads, $k=4$, with substitution token ratio ($r_s=50\%$).

D Methodology — Extra details

To assess BarcodeBERT's performance, we employed a combination of alignment-based methods, transformer-based models, and clustering algorithms. Here, we describe the parameters and hardware used in each methodology for reproducibility.

D.1 BLAST

We used BLAST [1] for species-level classification to align query sequences from the test subset of the *Seen* partition against a nucleotide database built from the training subset of the *Seen* partition. To ensure high-quality alignments, we restricted the output to the top-scoring hit per query and applied strict thresholds of at least 80% query coverage and 88% sequence identity. For genus-level classification, we used BLAST to align query sequences from *Unseen* partition against the same database built from the training subset of the *Seen* partition. We did not enforce a minimum coverage or sequence identity in this experiment as the expected sequence similarity at the genus level was lower than at the species level. We ran the experiments on an Intel(R) Xeon(R) CPU @ 2.20GHz using 4 CPU threads to maximize computational performance. The full commands used to run the experiments are as follows:

```
blastn -query supervised_test.fas -db train.fas
        -out results_supervised_top.tsv
        -max_target_seqs 1 -qcov_hsp_perc 80
        -perc_identity 88 -num_threads 4
        -outfmt 6
```

```
blastn -query unseen.fas -db train.fas
        -out results_unseen.tsv -max_target_seqs 1
        -num_threads 4 -outfmt 6
```

D.2 Baseline Models

For evaluation, we utilized the respective pretrained models from Hugging Face's ModelHub, specifically:

- DNABERT: github.com/jerryji1993/DNABERT
- DNABERT-2: huggingface.co/zhihan1996/DNABERT-2-117M
- DNABERT-S: huggingface.co/zhihan1996/DNABERT-S
- NT: huggingface.co/InstaDeepAI/nucleotide-transformer-v2-50m-multi-species
- HyenaDNA: huggingface.co/LongSafari/hyenadna-tiny-1k-seqlen-d256-hf

D.3 Model architecture configuration

To identify the optimal model configuration, we conducted a series of ablation experiments evaluating the impact of architectural choices, tokenization strategies, and the substitution component of the loss (w_s). We considered k-mer tokenization with four distinct k values (2, 4, 6, 8), as well as the BPE tokenizer. Three transformer configurations were evaluated: (i) 4 layers with 4 attention heads, (ii) 6 layers with 6 heads, and (iii) 12 layers with 12 heads. Additionally, we varied the penalty weight assigned to the substitution component of the loss (w_s) function, exploring values from 0.2 to 1.0.

Table S7 reports genus-level accuracy for 1-NN probing of unseen species across these settings. The best performance was obtained with the 4-4-4 configuration (4-mers, 4 heads, 4 layers), which we adopt as the default model throughout our evaluations.

D.4 Pretraining

BarcodeBERT was pretrained for 35 epochs using the AdamW optimizer [3] with a learning rate of $\alpha = 2 \times 10^{-4}$, a batch size of 128, and a OneCycle learning rate scheduler [6]. The pretraining process utilized four NVIDIA V100 GPUs and required approximately 36 hours to complete for each experiment executed. To examine the impact of pretraining, we also trained a model from scratch on the training subset of the *Seen* partition without any pretraining.

Table S7: Genus-level classification accuracy for 1-NN probing of unseen species using different k-mer sizes, transformer configurations, and substitution loss weights (w_s). Best results for each configuration are indicated in **boldface**.

	Genus-level acc (%) of unseen species with 1-NN probe														
	4 layers, 4 heads					6 layers, 6 heads					12 layers, 12 heads				
Loss weight (w_s)	$k=2$	$k=4$	$k=6$	$k=8$	BPE	$k=2$	$k=4$	$k=6$	$k=8$	BPE	$k=2$	$k=4$	$k=6$	$k=8$	BPE
0.2	64.18	76.06	75.15	71.15	70.57	61.59	74.61	70.87	67.74	67.15	48.92	63.72	57.12	56.40	62.34
0.5	66.47	74.98	76.62	71.22	70.34	65.38	73.37	70.87	69.70	67.57	46.23	67.11	61.24	60.05	62.20
0.8	68.84	76.71	74.66	73.33	69.40	68.37	74.02	72.20	69.72	68.23	60.50	67.50	66.87	61.05	67.09
1.0	76.92	**78.47**	75.74	75.62	69.85	67.71	73.91	74.38	**75.33**	70.45	**73.98**	68.27	68.67	73.79	68.16

D.5 Fine-tuning

All baseline models and BarcodeBERT were fine-tuned for 35 epochs on the supervised training subset of the *Seen* partition. For larger models, a batch size of 32 was used, while smaller models (CNN, HyenaDNA and BarcodeBERT) were trained with a batch size of 128. The AdamW optimizer [3] with a learning rate of $\alpha = 1 \times 10^{-4}$ was employed, coupled with the OneCycle learning rate scheduler [6]. We used a single NVIDIA V100 GPU for the fine-tuning process, completed within 18 hours for most models. Notably, HyenaDNA required significantly less time to fine-tune compared to other models, due to its lightweight architecture.

To train the fully-supervised BarcodeBERT model, which ablates the pretraining stage, we trained a randomly initialized model and then followed the same training procedure as our fine-tuning process.

D.6 Linear probe training

A linear classifier is applied to the embeddings generated by all the pretrained models for species-level classification. The models' parameters are learned using stochastic gradient descent with a constant learning rate of 1, momentum $\mu = 0.95$, and weight decay $\lambda = 1 \times 10^{-9}$.

D.7 Zero-shot clustering

We evaluated the models' ability to group sequences without supervision using a modified version of the framework from Lowe et. al. [4]. Embeddings were extracted from the pretrained encoders and reduced to 50 dimensions using UMAP [5] and cosine similarity to enhance computational efficiency while preserving data structure. These reduced embeddings were clustered with Agglomerative Clustering (L2 distance, Ward's linkage), using the number of true species as the target number of clusters. Clustering performance was assessed with adjusted mutual information (AMI) to measure alignment with ground-truth labels.

D.8 Additional classification metrics

To complement our species- and genus-level accuracy analyses, we also report precision, recall, and the weighted F1-score, which combines precision and recall together to create a single metric, computed (on our imbalanced barcode dataset) with sklearn's `average="weighted"` setting. The baselines include alignment-based BLAST, a non-SSL CNN encoder, and off-the-shelf DNA foundation models pretrained on generic genomic datasets. These are compared against BarcodeBERT (4-4-4), our model pretrained specifically on DNA barcode data.

Table S8: Precision score of different classification models under different evaluation strategies. Baselines are grouped into an alignment-based method (BLAST), a non-SSL CNN encoder, and pretrained DNA foundation models taken off-the-shelf. These are compared against BarcodeBERT (configured with $k=4$, 4 heads, 4 layers). Boldface indicates the **best result** per column, and underlines indicate <u>second place</u>.

Model	Species-level Precision (%) (seen species)	Species-level Precision (%) (linear probe)	Genus-level Precision (%) (1-NN probe)
BLAST	99.67	N/A	**84.61**
CNN encoder	98.38	N/A	57.02
DNABERT ($k=6$)	99.52	<u>98.83</u>	50.49
DNABERT-2	<u>99.72</u>	96.16	24.85
DNABERT-S	**99.73**	96.93	33.98
HyenaDNA-tiny (d256)	99.42	96.30	54.26
Nucleotide Transformer	99.47	96.84	43.88
**BarcodeBERT (4-4-4)**	99.62	**99.33**	<u>80.65</u>

Table S9: Recall score of different classification models under different evaluation strategies. Baselines are grouped into an alignment-based method (BLAST), a non-SSL CNN encoder, and pretrained DNA foundation models taken off-the-shelf. These are compared against BarcodeBERT (configured with $k=4$, 4 heads, 4 layers). Boldface indicates the **best result** per column, and underlines indicate <u>second place</u>.

Model	Species-level Recall (%) (seen species)	Species-level Recall (%) (linear probe)	Genus-level Recall (%) (1-NN probe)
BLAST	99.64	N/A	**83.88**
CNN encoder	98.07	N/A	54.43
DNABERT ($k=6$)	99.54	<u>98.50</u>	48.15
DNABERT-2	**99.81**	95.68	23.49
DNABERT-S	<u>99.72</u>	96.60	30.62
HyenaDNA-tiny (d256)	99.38	94.34	50.56
Nucleotide Transformer	99.62	96.11	40.11
**BarcodeBERT (4-4-4)**	99.57	**99.33**	<u>78.17</u>

Table S10: Weighted F1-scores of different classification models under different evaluation strategies. Baselines are grouped into an alignment-based method (BLAST), a non-SSL CNN encoder, and pretrained DNA foundation models taken off-the-shelf. These are compared against BarcodeBERT (configured with $k=4$, 4 heads, 4 layers). Boldface indicates the **best result** per column, and underlines indicate second place.

Model	Species-level F1 (%) (seen species)	Species-level F1 (%) (linear probe)	Genus-level F1 (%) (1-NN probe)
BLAST	99.66	N/A	**81.77**
CNN encoder	98.54	N/A	50.58
DNABERT ($k=6$)	99.53	98.48	45.09
DNABERT-2	99.70	95.61	21.59
DNABERT-S	**99.74**	96.85	48.00
HyenaDNA-tiny (d256)	99.12	96.21	47.98
Nucleotide Transformer	99.51	96.23	37.93
**BarcodeBERT (4-4-4)**	**99.74**	**99.34**	76.74

D.9 Additional performance metrics

Inference-time resource consumption for each model is summarized in Table S11. CPU consumption is broadly consistent across all the foundation models, with HyenaDNA using around 3% fewer resources than the other models. This state-space, attention-free model replaces quadratic self-attention with linear recurrence kernels, behaving like a CNN during inference and yielding minimal VRAM (∼130 MB) and host RAM (4.9 GB) consumption. In contrast, models in the DNABERT family require over 11 GB of system memory and 2.3–3.0 GB of GPU memory to store large attention matrices. Both the Nucleotide Transformer and BarcodeBERT moderate those costs through different design choices. NT uses the evolutionary-scale modelling architecture (7.6 GB RAM, 1.9 GB VRAM), and BarcodeBERT uses a compact BERT encoder (6.5 GB RAM, 0.7 GB VRAM). BLAST remains the most efficient option, with zero VRAM usage and only 222 MB of host RAM, reflecting its disk-backed, optimized indexing engine. Future work may include model compression (e.g. pruning, quantization) and optimized inference kernels to further narrow the efficiency gap among deep learning approaches.

Model	CPU utilization (%)	RAM (MB)	VRAM (MB)
BLAST	7.26	222	0
CNN encoder	22.11	5,332	98
DNABERT ($k=6$)	24.91	11,345	2,285
DNABERT-2	25.01	12,669	3,010
DNABERT-S	25.01	12,696	3,009
HyenaDNA-tiny (d256)	22.18	4,851	131
Nucleotide Transformer	24.96	7,607	1,885
BarcodeBERT	25.42	6,493	694

Table S11: Resource usage comparison of DNA models. CPU usage is normalized to a 4-core system.

References

[1] Stephen F Altschul et al. "Basic Local Alignment Search Tool". In: *J. Mol. Biol.* 215.3 (1990), pp. 403–410. DOI: 10.1016/S0022-2836(05)80360-2.

[2] Jacob Devlin et al. "BERT: Pre-training of Deep Bidirectional Transformers for Language Understanding". In: *Proceedings of the 2019 Conference of the North American Chapter of the Association for Computational Linguistics: Human Language Technologies, Volume 1 (Long and Short Papers)*. Minneapolis, Minnesota: Association for Computational Linguistics, June 2019, pp. 4171–4186. DOI: 10.18653/v1/N19-1423.

[3] Ilya Loshchilov and Frank Hutter. "Decoupled Weight Decay Regularization". In: *ICLR*. 2017. URL: https://openreview.net/forum?id=Bkg6RiCqY7.

[4] Scott C. Lowe et al. "An Empirical Study into Clustering of Unseen Datasets with Self-Supervised Encoders". In: *arXiv preprint* (2024). DOI: 10.48550/arxiv.2406.02465. arXiv: 2406.02465.

[5] Leland McInnes, John Healy, and James Melville. "UMAP: Uniform manifold approximation and projection for dimension reduction". In: *arXiv preprint* (2018). DOI: 10.48550/arxiv.1802.03426. arXiv: 1802.03426.

[6] Leslie N. Smith and Nicholay Topin. "Super-Convergence: Very Fast Training of Residual Networks Using Large Learning Rates". In: *arXiv preprint* (2017). DOI: 10.48550/arxiv.1708.07120. arXiv: 1708.07120.
